# Magnesium and Cardiac Arrythmias: An Overlooked Guardian of Electrical Stability

**DOI:** 10.14740/cr2198

**Published:** 2026-04-15

**Authors:** Kaiyu Jia, Joaquim Anthony Noguer, Elizabeth R. Rimsky, Gauri Shailesh Pikale, Aysan Sattarzadeh, Arun Gajan Pradeep, Omar Khayat, Kashif Ahmad, Dov Vachss, Felicia Zhang, Esther Pearce, Shahkar Khan, Suzanne El-Sayegh

**Affiliations:** a Department of Medicine, Staten Island University Hospital, Staten Island, NY, USA; b Department of Medicine, Northwestern Medicine McHenry Hospital, McHenry, IL, USA; c Department of Cardiology, Staten Island University Hospital, Northwell Health, NY, USA; d Department of Nephrology, Staten Island University Hospital, Northwell Health, NY, USA

**Keywords:** Magnesium, Hypomagnesemia, Cardiac arrhythmias, Mechanism, Treatment

## Abstract

Magnesium is one of the inconspicuous cations involved in multiple systems of the human body. Yet, laboratory evaluation of this ion is not routinely conducted. It plays a crucial role in the cardiac system, and abnormal levels can lead to arrhythmias by disrupting the flow of other ions involved in the cardiac membranes. This review aimed to highlight the common etiologies of hypomagnesemia and the fundamental roles of magnesium in the body with particular attention to the mechanism of action of magnesium related to suppression and termination of cardiac arrhythmias, as well as its crucial roles in the treatment of ventricular arrhythmias and reperfusion injuries after myocardial infarction.

## Introduction

Cardiac arrhythmia is characterized based on the heart rate and rhythm as tachycardic or bradycardic and regular, regularly irregular or irregularly irregular, respectively. Tachycardia is defined as a heart rate (HR) > 100 beats per minute (bpm). Bradycardia refers to HR < 60 bpm. Symptoms of cardiac arrhythmia include dizziness, palpitations, weakness and shortness of breath. Signs of arrhythmia include loss of consciousness, observation of irregular neck vein pulsation, and palpation of an irregular pulse. The diagnosis of arrhythmia is established based on vital signs and electrocardiography (ECG). Event or Holter monitors can be used to facilitate diagnosis when the arrhythmia is suspected but not observed, such as in the case of paroxysmal, or intermittent, arrhythmias. Arrhythmias can lead to multiple complications, the most dramatic being sudden cardiac death (SCD) [1].

SCD is defined as death from cardiovascular causes within 1 h of symptom onset and can result from a variety of conditions. When toxicological and pathological screens are negative, this condition is called “sudden arrhythmic death syndrome (SADS)” [2]. Given the futility of treating a patient for SCD, the focus remains on SCD prevention. While there are numerous causes of SCD, the underlying mechanism in 84% of cases is a fatal ventricular arrhythmia, mostly ventricular tachycardia or fibrillation. Less common causes of SCD are bradycardia and pulseless electrical activity (PEA).

Ventricular arrhythmias are defined as wide-complex rhythms that may be regular or irregular, including ventricular tachycardia, ventricular fibrillation, and premature ventricular contractions. Underlying causes of ventricular arrhythmias include structural causes as well as non-structural causes. Structural causes or ischemic causes such as coronary artery disease, particularly in the setting of prior myocardial infarction with resultant myocardial scarring, accounts for a majority of SCD cases due to the formation of arrhythmogenic reentry circuits [3, 4]. Other structural causes include dilated cardiomyopathy, hypertrophic cardiomyopathy, arrhythmogenic right ventricular cardiomyopathy, congenital heart disease, valvular heart disease, myocarditis, infiltrative cardiomyopathies such as cardiac sarcoidosis, congenital heart disease, and long-standing hypertension leading to anatomical variations or structural myocardial remodeling [3, 4]. In contrast, non-structural causes often involve channelopathies or abnormalities or conduction pathways. These conditions include inherited channelopathies such as long QT syndrome, Brugada syndrome, and catecholaminergic polymorphic ventricular tachycardia (CPVT), accessory pathway-mediated arrhythmias such as Wolff-Parkinson-White (WPW) syndrome, which predispose patients to malignant tachyarrhythmias in the absence of structural heart disease [3, 4].

One of the frequently overlooked causes of SCD is electrolyte abnormalities. Electrolyte imbalance is common in patients with chronic heart failure, especially those managed with diuretics. Both loop and thiazide diuretics can result in electrolyte disturbances with hypokalemia and hypomagnesemia being most common. In patients already predisposed to SCD, these electrolyte disturbances can result in fatal arrhythmias. This highlights the imperative need to monitor serum electrolytes frequently in patients at increased risk of SCD due to drug-induced electrolyte abnormalities [4]. Importantly, these electrolyte disturbances (i.e., hypo- and hyperkalemia, hypomagnesemia, hypocalcemia, hyponatremia. etc.) exert their proarrhythmic effects largely through alterations in cardiac ion channel activity and membrane potentials. These abnormalities in ionic currents can disrupt normal cardiac depolarization and repolarization processes, predisposing patients to ventricular and/or atrial arrhythmias.

## Cardiac Membrane Ion Channels and Electrolytes Involved

The significant ions during depolarization and contraction are sodium and calcium (Ca^2+^) ions. Once contraction occurs, the membranes of the cardiac muscle spontaneously revert to the resting membrane potential (RMP) with the help of efflux of potassium (K^+^) [5].

To understand the implications of these ion channels, we must first understand how and where they function. The primary ion channels participating in the cardiac membrane potential are as follows [6]. Voltage-gated sodium channels are responsible for depolarization of the membrane once the threshold has been reached. Voltage-gated Ca^2+^ channels are responsible for the contraction of the myocyte once the threshold voltage is achieved. Voltage-gated K^+^ channels are responsible for the outward current required to repolarize the cardiac cell. Inward rectifying K^+^ channels are a small group of ion channels responsible for cardiac membrane stabilization. Rectification is the ability of an object or material to allow the flow of current in only one direction. Inward and outward refers to the direction of current flow. Inward rectification allows current to flow inside the cell, and outward rectification allows current to flow outside the cell.

The Kir2 family of mammalian K^+^ inward rectifiers have five strongly rectifying members. Kir2.1, the first identified, is the major ion channel responsible for the I_K1_ current (i.e., the current during the inward rectification of K^+^ via Kir2 channels) in ventricular myocytes and is a crucial regulator of RMP [7–9]. Magnesium (Mg^2+^) and other polyamines act together to cause robust inward rectification of Kir2.1 currents and I_K1_. The predominant channel of this subset in atrial myocytes is the Kir2.3 subset, and studies have documented that I_K1_ channels may be part of the similarly related subunits Kir2.1, Kir2.2 and Kir2.3 [10, 11].

## Mechanism of Hypomagnesemia-Induced Cardiac Arrhythmias

The renal outer medullary potassium (ROMK) channels are inwardly rectifying K^+^ channels [12]. This channel is the first member of the Kir subset of inwardly rectifying K^+^ channels and is also known as Kir2.1. These channels are blocked by Mg^2+^ and various other polyamines. In states of normal Mg^2+^ levels, these channels only allow K^+^ to move inward into cells and help in the renal ability to conserve K^+^. These channels allow K^+^ to move outward freely in states of low Mg^2+^ levels, resulting in loss of K^+^ into the tubular lumen and urine. Mg^2+^ ions bind to the intracellular domain of these channels and prevent the efflux of K^+^ from cells. In a state of low Mg^2+^, excessive K^+^ wasting occurs in the kidney, even if hypokalemia is present. This can exacerbate hypokalemia or even precipitate hypokalemia. Supplemental K^+^ will not correct this abnormality, and in cases like this correcting the Mg^2+^ levels will correct the hypokalemia by allowing the kidney to reabsorb K^+^ adequately [12–14].

The inward rectifying K^+^ channels (such as Kir2.1) are a unique subset of K^+^ channels that allow potassium to move more easily into the cell than out of the cell, contributing in stabilizing the RMP and contributing to the final phase of repolarization of the cardiac action potential [15]. Hyperpolarization of the cardiac cell is a unique state during which K^+^ ions can flow inward through the inward rectifying channels. Our next question would be, what gives these unique K^+^ channels the ability to rectify? The answer is that Mg^2+^ ions and polyamines (i.e., small positively charged organic molecules naturally present inside myocardium cells that interact with DNA, proteins, and ion channels such as putrescine, spermidine, and spermine) block this channel during other phases of cardiac potentials; thus, these channels play an important role in cardiac membrane stabilization as well as giving its “inward rectification” characteristics. Mg^2+^ ions grant these channels the ability to rectify; thus, Mg^2+^ will be our focus for the rest of the article. Mg^2+^ ions block these channels on the intracellular side and prevent the efflux of K^+^ ions during the depolarization phase, allowing plateau potential to be attained. During phases of highly negative cardiac potentials, they allow for the inward flow of K^+^ ions. Unfortunately, no manufactured or biological system is perfect, and in conditions of hypomagnesemia, these rectifiers do not perform their function appropriately, creating conditions that result in abnormal cardiac membrane electrical activities, and hence arrhythmias may develop [15, 16].

## Causes of Hypomagnesemia

Hypomagnesemia is defined as Mg^2+^ level of less than 1.7 mg/dL (1.4 mEq/L or 0.7 mmol/L). While chronic alcohol usage is the most common cause, other common causes of hypomagnesemia include vomiting, loop diuretics, thiazide diuretics, proton pump inhibitors (PPIs), low dietary intake, diarrhea, refeeding syndrome, acute pancreatitis due to saponification, osmotic diuresis, and genetic renal Mg^2+^ handling abnormalities [17–20].

The clinical manifestations of hypomagnesemia can vary widely from nonspecific signs and symptoms such as muscle cramps, weakness, paraesthesia to life-threatening symptoms such as seizures, neuromuscular weakness, tetany and arrhythmias including ventricular fibrillation and torsade de pointes [21].

An important topic to touch upon for this review is hypokalemia due to hypomagnesemia. This is majorly related to the ROMK channels in the kidney, a similar group of inwardly rectifying K^+^ channels also found in the heart [22].

## Role of Mg^2+^ in the Treatment of Cardiac Arrhythmias

Mg^2+^ is predominantly present intracellularly as a divalent cation. It is an essential mineral, serves as a cofactor for various enzyme systems, and plays an integral role in several biochemical reactions in the body [23]. Mg^2+^ deficiency has been implicated in a large variety of disease processes, including, but not limited to, migraines, Alzheimer’s disease, cerebrovascular accidents, hypertension, cardiovascular diseases, and type 2 diabetes mellitus [24]. Hypomagnesemia can result in electrophysiological disturbances that can present as short PR and QRS and, to a lesser degree, and prolongation of QT interval. Severe hypomagnesemia can manifest as tachycardia that is commonly followed by ventricular arrhythmia and, eventually, bradycardia.

Mg^2+^ has several functions in the cardiovascular system by acting on various ion channels present in the cardiac cells, especially the Ca^2+^ and K^+^ channels and Na/K ATPase in the cardiac contractile tissue and pacemaker cells [25].

The anti-arrhythmic effect of Mg^2+^ can be attributed to its ability to regulate intracellular K^+^ and Ca^2+^ concentrations. It maintains intracellular K^+^ concentration, thus reducing the likelihood of repolarization. K^+^ influx is responsible for phase 3, the repolarization phase of the cardiac action potential. It also acts as a Ca^2+^ antagonist by binding to the high-affinity site for Ca^2+^ on actin, which helps in improving diastolic relaxation. It modulates Ca^2+^ handling in the sarcoplasmic reticulum [26, 27]. Many studies indicate that the I_K1_ transient caused by the assistance of the Mg^2+^ block can substantially enhance the “cardiac repolarization reserve,” and subsequently protect against arrhythmia [28].

Mg^2+^ is the drug of choice in treating torsade de pointes, which can be precipitated by a wide variety of drug classes that lead to QTc prolongation [29–31]. Mg^2+^ decreases the early after-depolarization amplitude and prevents it from reaching the threshold. Magnesium sulfate tetrahydrate, 2 g, is given as an intravenous (IV) infusion over 10–15 min, and the plasma levels must be monitored for toxicity [32].

Mg^2+^ is also an effective treatment modality in digitalis-induced arrhythmias. It is used in acute digitalis poisoning and prevents the efflux of K^+^ caused by digitalis. The dosage is 2 g 10% magnesium sulfate infused intravenously over 20 min [33].

The use of Mg^2+^ in myocardial infarction patients have been previously investigated in large-scale studies. Although evidence suggests that Mg^2+^ can improve survival in patients with myocardial infarction by preventing arrhythmias [34], the Fourth International Study of Infarct Survival (ISIS-4) study did not demonstrate a significant short-term mortality benefit of Mg^2+^ in patients with acute myocardial infarction [35]. Also, IV magnesium sulfate infusion has been shown to reduce the occurrence of fatal ventricular arrhythmias after open heart surgery by reducing ventricular excitability [36]. This discrepancy could be attributed to multiple confounding factors such as comorbidities, extent of myocardial infarction, as well as underlying types of arrhythmias.

The role of Mg^2+^ in atrial arrhythmias is not well defined. Studies performed in human hearts have revealed that Mg^2+^ increases refractoriness and atrial conduction times [37]. These point toward the potential use of Mg^2+^ in treating atrial arrhythmias. One of the studies has demonstrated a significant reduction in the incidence of atrial fibrillation (AF) in patients who received magnesium sulfate prophylaxis after coronary artery grafting [38]. Few other studies have reported that Mg^2+^ has no clinical or statistical effect on preventing atrial arrhythmias [39, 40].

Mg^2+^ has also proved beneficial in reducing reperfusion injury after ischemia by attenuating the inflammatory response [41]. An interesting and important point to note is that IV magnesium sulfate is ineffective in monomorphic ventricular tachycardia and shock-resistant ventricular fibrillation [42].

Some other conditions where Mg^2+^ is used include conditions predisposing to AF, prevention of AF in patients undergoing cardiac and thoracic surgery, and treatment of AF by reducing rapid ventricular response. Mg^2+^ can also be used to revert polyventricular tachycardia (PVT) to sinus rhythm [42].

## Limitations and Adverse Reactions

Normal serum Mg^2+^ levels vary in existing literature and generally fall within the range of 1.4–2.0 mEq/L [43–45]. Mg^2+^ is administered orally, intramuscularly, intraosseously, or intravenously. The most common preparation used is magnesium sulfate, which contains 98.6 mg or 8.12 Eq for every 1 g of magnesium sulfate. This makes magnesium sulfate a relatively safe drug to administer when indicated as the therapeutic index is broad. However, for symptoms of toxicity to occur, one will have to infuse vast amounts of the drug erroneously [46].

One of the major reasons for Mg^2+^ toxicity is renal dysfunction, as Mg^2+^ is renally excreted. Another cause of Mg^2+^ toxicity is cellular lysis in chemotherapy patients, as Mg^2+^ concentrations are much higher intracellularly [47].

Mg^2+^ toxicity initially can present with nonspecific symptoms, but the loss of patellar reflexes is the most characteristic, which occurs at approximately 8–10 mEq/L. With increasing concentrations, there is respiratory depression and paralysis between 10–15 mEq/L and finally cardiac arrest at levels close to 25 mEq/L. From this, it is evident that one has to make a gross error in administration for serious harm to occur in a patient [46]. Previous case reports regarding Mg^2+^ toxicity have reported Mg^2+^ levels above 16 mmol/L due to iatrogenic errors of magnesium sulfate infusion [48, 49].

Contraindications to administering magnesium sulfate include renal impairment, especially if creatinine clearance is < 30 mL/min [19]. In addition, Mg^2+^ is avoided in myasthenia gravis as Mg^2+^ inhibits the release of acetylcholine from the neuromuscular junction [50, 51].

In the rare cases of severe Mg^2+^ toxicity, there are multiple treatment options available depending on the presentation. In mild toxicity, one needs to discontinue any over-the-counter Mg^2+^ supplementation. In severe toxicity with respiratory depression, ventilatory support may be needed. In severe toxicity, saline diuresis and furosemide are tried if renal function is adequate. If symptoms of cardiac toxicity are predominant, then IV calcium gluconate/calcium chloride may be used. Finally, renal dialysis may be indicated for refractory symptoms of severe toxicity [52–55].

Of interest to note is that calcium gluconate or chloride is a highly effective drug to counteract the effects of hypermagnesemia, as Mg^2+^ directly and indirectly inhibits Ca^2+^ and K^+^ channels. Hence the administration of calcium reverses these effects [56].

## Conclusions

This review highlights the role of Mg^2+^, as well as its normal serum levels, in the prevention of cardiac arrhythmias. Hypomagnesemia, intertwined with hypokalemia, is an arrhythmogenic electrolyte disturbance. In some conditions, magnesium sulfate is used therapeutically to suppress cardiac arrhythmias. Neglecting serum Mg^2+^ levels can lead to fatal cardiac arrhythmias especially in patients with cardiac comorbidities. Nonetheless, magnesium sulfate is not included in the conventional list of antiarrhythmics as no large-scale randomized controlled trials have yet to definitively demonstrated its efficacy for certain types of arrhythmias, especially atrial arrhythmias, in reducing arrhythmia incidence, improving rhythm control, or lowering arrhythmia-related morbidity and mortality. The authors aim to highlight Mg^2+^ as a cation commonly overlooked and underscore the need for large studies to demonstrate the benefits of magnesium sulfate in the suppression and termination of cardiac arrhythmias.

## Data Availability

The authors declare that data supporting the findings of this study are available within the article.

## Abbreviations

AF: atrial fibrillation
bpm: beats per minute
CPVT: catecholaminergic polymorphic ventricular tachycardia
Ca^2+^: calcium
ECG: electrocardiography
HR: heart rate
Mg^2+^: magnesium
PVT: polyventricular tachycardia
K^+^: potassium
PPI: proton pump inhibitor
PEA: pulseless electrical activity
ROMK: renal outer medullary potassium
RMP: resting membrane potential
SADS: sudden arrhythmic death syndrome
SCD: sudden cardiac death
WPW: Wolff-Parkinson-White
